# *MT-CYB* mutations in hypertrophic cardiomyopathy

**DOI:** 10.1002/mgg3.5

**Published:** 2013-04-12

**Authors:** Christian M Hagen, Frederik H Aidt, Ole Havndrup, Paula L Hedley, Cathrine Jespersgaard, Morten Jensen, Jørgen K Kanters, Johanna C Moolman-Smook, Daniel V Møller, Henning Bundgaard, Michael Christiansen

**Affiliations:** 1 Department of Clinical Biochemistry, Immunology, and Genetics, Statens Serum InstitutCopenhagen, Denmark; 2 Department of Biomedical Sciences, University of CopenhagenCopenhagen, Denmark; 3 Institute of Cellular and Molecular Medicine, Faculty of Health Sciences, University of CopenhagenCopenhagen, Denmark; 4 Department of Cardiology, Roskilde SygehusRoskilde, Denmark; 5 Department of Biomedical Sciences, Stellenbosch UniversityCape Town, South Africa; 6 Department of Medicine B, The Heart Center, RigshospitaletCopenhagen, Denmark

**Keywords:** Cardiomyopathy, DNA sequencing, genetic disorders, hypertrophy, mitochondria

## Abstract

Mitochondrial dysfunction is a characteristic of heart failure. Mutations in mitochondrial DNA, particularly in *MT-CYB* coding for cytochrome B in complex III (CIII), have been associated with isolated hypertrophic cardiomyopathy (HCM). We hypothesized that *MT-CYB* mutations might play an important causal or modifying role in HCM. The *MT-CYB* gene was sequenced from DNA isolated from blood from 91 Danish HCM probands. Nonsynonymous variants were analyzed by bioinformatics, molecular modeling and simulation. Two germline-inherited, putative disease-causing, nonsynonymous variants: m.15024G>A; p.C93Y and m.15482T>C; p.S246P were identified. Modeling showed that the p.C93Y mutation leads to disruption of the tertiary structure of Cytb by helix displacement, interfering with protein–heme interaction. The p.S246P mutation induces a diproline structure, which alters local secondary structure and induces a kink in the protein backbone, interfering with macromolecular interactions. These molecular effects are compatible with a leaky phenotype, that is, limited but progressive mitochondrial dysfunction. In conclusion, we find that rare, putative leaky mtDNA variants in *MT-CYB* can be identified in a cohort of HCM patients. We propose that further patients with HCM should be examined for mutations in *MT-CYB* in order to clarify the role of these variants.

## Introduction

Hypertrophic cardiomyopathy (HCM) is characterized by hypertrophy of the left ventricle, predominantly the interventricular septum. The clinical manifestations are dyspnoea, angina, palpitations, syncope, and an increased propensity for sudden arrhythmic death (Elliott et al. 2008b). Microscopically, the disease is characterized by myocyte disarray and fibrosis (Elliott et al. 2008b). HCM has, during the last two decades, been shown to be largely a genetic disease caused by mutations in genes encoding proteins of the sarcomere, but despite the identification of mutations in 13 genes causing HCM, no genetic etiology have been found in 30–50% of cases (Andersen et al. 2009). The disease has a prevalence of 1:500 (Maron et al. 1987) and a highly variable clinical presentation. Some mutation carriers develop disease and die in early childhood (Ostman-Smith et al. 2008), whereas others exhibit late onset disease or remain asymptomatic (Elliott et al. 2008b).

Mitochondrial function is essential for cardiomyocyte function (Rosca and Hoppel 2010). The mitochondrial proteome consists of at least 1500 proteins of which 13 are encoded by the mitochondrial DNA (mtDNA) (Taylor et al. 2003). Furthermore, mtDNA also encodes 22 tRNAs and two rRNAs. Mitochondrial oxidative phosphorylation (OXPHOS) is performed by five large multisubunit complexes (I–V) (Scheffler 2008).

A number of mutations in mtDNA as well as nuclear genes involved in mitochondrial function have been shown to cause HCM, both as an isolated phenotype and as part of complex syndromes (Andreu et al. 2000; Moslemi et al. 2008; http://mitomap.org 2012). Several pathogenic mechanisms have been described, causing reduced ATP production as well as increased reactive oxygen species (ROS) production and aberrant apoptotic induction (Tuppen et al. 2010). It has been reported that at least 1:200 unselected newborns harbor mtDNA mutations that could potentially cause disease (Elliott et al. 2008a). Mitochondrial mutations can be divided into two types: severe heteroplasmic mutations, such as m.3243A>G, causing the mitochondrial encephalomyopathy, lactic acidosis, and stroke-like episodes (MELAS) syndrome (Nishino et al. 1996), and the usually homoplasmic leaky mutations, with incomplete penetrance, varying age-of-onset and severity, such as the m.1555A>G mutation predisposing for deafness (Matsunaga et al. 2004). Furthermore, mtDNA variants are difficult to interpret because of the extensive sequence variation (Montoya et al. 2009), the complex genotype–phenotype relationship and the difficulty in performing functional analyses on the mutated proteins (Tuppen et al. 2010).

A particularly interesting HCM mtDNA candidate gene is *MT-CYB,* coding for cytochrome b (Cytb), a component of complex III (CIII). Missense mutations in this gene have been associated with several diseases with cardiac and muscular involvement, including HCM (Valnot et al. 1999; Andreu et al. 2000; http://mitomap.org 2012). Despite this, the role of *MT-CYB* mutations in the causation or phenotypic modification of HCM has not yet been clarified in a large group of consecutively collected HCM patients already screened for mutations in established HCM-associated genes.

Hence, the purpose of this study was to examine the role of variations in *MT-CYB* in the causation of HCM in a large HCM cohort. We screened 91 well-characterized HCM probands for mutations in *MT-CYB* and analyzed the potential significance of the variants identified.

## Materials and Methods

### Ethics statement

Written informed consent was obtained from study participants. The study was approved by the Local Science Ethics Committees, Copenhagen and Frederiksberg, protocol no. KF V92213. The control samples from anonymous Danish blood donors were obtained from Rigshospitalet, Copenhagen. The blood donors gave written informed consent.

### Patients

Ninety-one unrelated, consecutively diagnosed HCM patients identified at, or referred to, Copenhagen University Hospital, The Heart Centre, Rigshospitalet, Copenhagen, Denmark were included in the study. Patients were subjected to a full clinical evaluation including family history, physical examination, echocardiography, and electrocardiography. All fulfilled classical diagnostic criteria for familial HCM (ten Cate et al. 1979; Richardson et al. 1996). None of the patients had elevated serum lactate or suffered from hearing loss. Forty-eight percent were familial cases. A summary of demographic and clinical characteristics of the patient cohort is given in Table 1. Ninety-four percent had septal hypertrophy and 6% had apical hypertrophy. All patients had been screened for mutations in the coding regions of *MYH7*, *MYBPC3*, *TTNT2*, *TPM1*, *TNNI3*, *MYL3*, *MYL2*, *ACTC*, *TCAP*, *CSRP3*, *CRYAB*, and exons 3, 7, 14, 18, and 49 of *TTN*, as detailed in a previous study (Andersen et al. 2009). All patients were also screened for mutations in *GLA*. In 32 patients, this screening had identified what appeared to be disease-causing mutations, viz, 12 in *MYH7*, eight in *MYBPC3*, two in each of *TNNT2*, *TNNI3*, and *GLA*, one in each of *ACTC*, *TPM1*, *MYL3*, and *MYL2*. Two patients were carriers of mutations in both *MYL2* and *MYH7*.

**Table 1:** Demographic and clinical characteristics in the 91 HCM probands

Parameter	Mean (SD)	Parameter	Mean (SD)
Age (years)	49 (16)	LVEDD (mm)^2^	47 (8)
Men/Women	154/37	LVESD (mm)^2^	28 (10)
BP systolic (mmHg)	128 (21)	LVIVS(middle) (mm)	17 (5)
BP diastolic (mmHg)	76 (14)	LVIVS(Ivot) (mm)^2^	20 (5)
LA (mm)^2^	45 (9)	LVPWT (mm)^2^	12 (4)
Max LVD (mm)^2^	20 (6)	EF (%)	68 (16)
Max IVS (mm)^2^	20 (5)	LVIRT (sec)	0.081 (0.023)
BMI (kg/m^2^)	25 (4)		

HCM, hypertrophic cardiomyopathy; BP, blood pressure; LA, left atrial diameter; MaxLVD, maximal left ventricular wall thickness; MaxIVS, maximal interventricular wall thickness; LVEDD, left ventricular end diastolic diameter; LVSD, left ventricular systolic diameter; LVIVS, left ventricular interventricular septal thickness; LVPWT, left ventricular posterior wall thickness; EF, ejection fraction; LVIRT, left ventricular isovolumic relaxation time.

1 Actual numbers.

2 Measured in index patients >18 years of age.

### Mutation screening of MT-CYB

DNA was extracted from blood using the Maxwell® 16 System (Promega Corporation, Madison, WI). All primers were designed using the Reversed Cambridge sequence (rCRS, GenBank ID: NC_012920) as template DNA for amplification of *MT-CYB* (position 14747-15887). All PCR reactions were performed at an annealing temperature of 60°C. The PCR products were sequenced using a BigDye Terminator v1.1 Cycle Resequencing Kit (Applied Biosystems, Life Technologies Corporation, Carlsbad, CA), and analyzed on an ABI3730 DNA Analyzer. The resulting sequences were aligned and compared to the rCRS using Sequencher 4.8 software (Gene Codes, Ann Arbor, MI). The heteroplasmic levels of probands B and I, carrying mutation m.15024G>A and m.15482T>C, respectively, were quantified using pyrosequencing in all maternal relatives; the assay was designed with PyroMark Assay Design Software 2.0 and was executed on a Pyrosequencer PSQ 96MA (Qiagen, Hilden, Germany). All primer sequences are available on request.

### Criteria for putative disease causation

Synonymous mutations were considered noncausative. Nonsynonymous mutations with a database frequency ≥0.1% were considered unlikely to be disease causing. Database frequency was established from GenBank, RefSeq, EMBL, DDBJ, and Protein Data Bank databases using the following entrez query: “Homo”[Organism] AND mitochondrion[FILT] NOT {(“Homo sp. Altai”[Organism] OR “Denisova hominin”[ALL]) OR “neanderthalensis”[ALL]}. Due to the high mutation rate in mitochondrial DNA, we chose a frequency of ≥0.1% rather than the usual distinction between polymorphisms and mutations in nuclear genes of 1%. The remaining nonsynonymous mutations were considered putative disease causing if modeling supported a significant effect upon protein function, if it interfered with an evolutionary conserved amino acid, or if there was evidence in the literature of disease causation. If a variant defined multiple subclades in PhyloTree-mtDNA Tree Build 15 (30 September 2012), or was a characteristic marker of an older clade, it was considered nonpathogenic. Furthermore, the reported status of the variants identified in the MitoMap database (http://www.mitomap.org) was taken in consideration. The frequency of mutations considered likely disease causing was established by screening a local population of 192 Danish anonymous samples obtained from the blood bank at Rigshospitalet, Copenhagen and 288 commercially obtained British Caucasian samples (ECACC, Salisbury, U.K.).

### Bioinformatics

The consequence of the variants was established using MitoWheel (http://mitomap.org). All nonsynonymous variants were searched for in the MitoMap database, OMIM (http://www.omim.org), PhyloTree (http://www.phylotree.org), and Google Scholar. Variants not found by any of the methods listed above were searched for in GenBank, RefSeq, EMBL, DDBJ, and PDB using Geneious 4.8 (Biomatters Ltd, Auckland, New Zealand) in order to identify nonpublished variants. All variants were submitted to PolyPhen (http://genetics.bwh.harvard.edu/pph/) using the entire sequence of Cytb with the relevant amino acid substituted (UniProt P00156). Multiple alignment and conservation scores were generated using ClustalX (http://www.clustal.org) and Jalview (http://www.jalview.org).

### Molecular modeling

Putative disease-causing variants were visualized using Swiss-PDB Viewer v.4.0.2 (http://spdbv.vital-it.ch/). As the crystal structure of human mitochondrial cytochrome bc_1_ complex has not yet been solved, the homologue-based structural prediction service I-TASSER (Roy et al. 2010) (http://zhang.bioinformatics.ku.edu/I-TASSER/) was used to generate the structure by submitting the human Cytb sequence (UniProt: P00156). The resulting structure was aligned with the crystal structure of *Bos Taurus* mitochondrial cytochrome bc1 complex (PDB ID: 1ntz). Using Swiss-PDB Viewer, amino acid changes were introduced into the structure and all possible rotamers were evaluated. Energy minimization was applied, followed by analysis using the implementation of the GROMOS 43B1 force field, together with putative H-bond changes. The yeast CIII (pdb:3CXH) was used as a homologue in analyzing macromolecular interactions.

### Molecular dynamics

Molecular dynamics was assessed using the NAMD program (Phillips et al. 2005) and the CHARMM22 force field (MacKerell et al. 1998). The protein coordinates generated from I-TASSER were used as a template for the construction of a set of starting coordinates, which were used to relax the system into a production-ready stage. The computation was done on a water-dissolved protein, and the simulation was run for either 10 or 20 nsec under spherical boundary conditions, with rigid water H-bonds, using langevin dynamics and a constant temperature of 310 K. The simulation was run on a CUDA-accelerated high-performance computer. VMD 1.9 was used to analyze trajectories and generate Ramachandran plots (Humphrey et al. 1996; http://www.ks.uiuc.edu/Research/vmd/). Protein secondary structure was generated from the trajectories using STRIDE (Frishman and Argos 1995).

## Results

**Figure 1:**
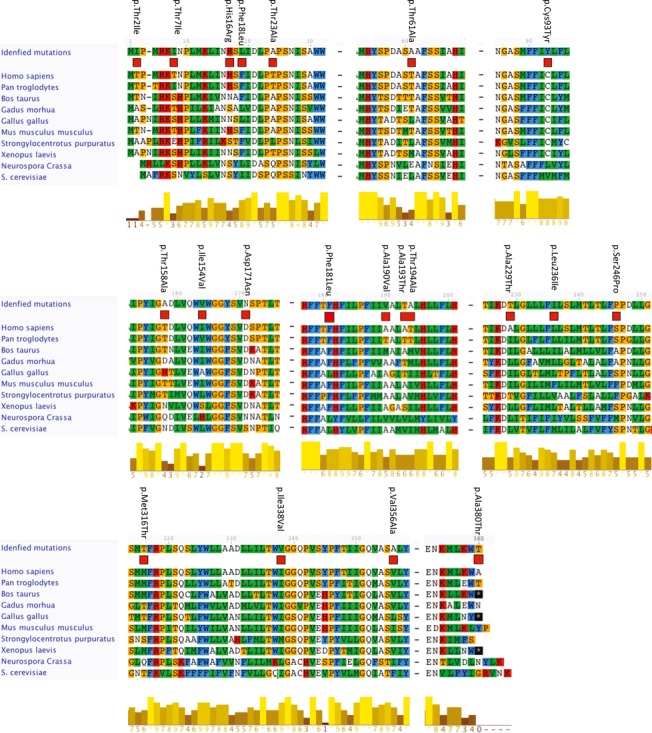
Cytb conservation index. Multiple alignments of the Cytb amino acid sequences with the identified nonsynonymous mutations marked. The position of the mutation is highlighted and the amino acids are numbered according to the *Homo sapiens* sequence. The conservation scores are generated by Jalview and reflect the conservation of the physicochemical properties of each column in the alignment.

Forty-three deviations from the rCRS were identified in the 91 probands. Twenty-one of these were nonsynonymous (shown in Tables 2 and 3). The conservation of the involved amino acids is shown in Figure 1. Eleven variants were excluded according to our frequency criteria and/or PhyloTree status; these were consequently considered polymorphisms (Table 2). The remaining 10 were analyzed further (Table 3). Two variants were found to be putative disease causing.

**Table 2:** Identified nonsynonymous variants with a database frequency >0.1%

Bp variant	AA no. substitution	MitoMap status^1^	Frequency (%)	Frequency HCM patients
m.14766C>T	p.T7I	Polymorphism	∼70.4	33.3
m.14793A>G	p.H16R	Polymorphism	∼2.3	6.6
m.14798T>C	p.F18L	Polymorphism	∼8.7	6.6
m.15218A>G	p.T158A	Polymorphism	∼1.9	2.2
m.15236A>G	p.I164V	Polymorphism	∼0.8	1.1
m.15323G>A	p.A193T	Polymorphism	∼0.5	1.1
m.15326A>G	p.T194A	Polymorphism	∼96.8	100
m.15431G>A	p.A229T	Polymorphism	∼1.3	1.1
m.15693T>C	p.M316T	Polymorphism/Possibly LVNC-associated	∼1.3	2.2
m.15758A>G	p.I338V	Polymorphism	∼0.8	1.1
m.15884G>A	p.A380T	Polymorphism	∼1.0	2.2

1 MitoMap status: current status of the variant in the MitoMap database (http://www.mitomap.org).

**Table 3:** Identified nonsynonymous variants with a population frequency <0.1% or variants which have been previously associated with HCM

Bp variant	AA no. substitution	MitoMap status^1^	Frequency (%)	Frequency HCM patients.	Conservation score (Fig. 1).	Indication of structural effect using molecular modeling	References
m.14751C>T	p.T2I	Polymorphism	∼0.05	1.1	1	Unlikely	Hinttala et al. (2006)
m.14813A>G	p.T23A	Not present	∼0.01	1.1	5	Unlikely	Behar et al. (2008)
m.14927A>G	p.T61A	Polymorphism	∼0.6	1.1	4	Unlikely	Casademont and Miro (2002); Marin-Garcia et al. (2000); Obayashi et al. (1992)
m.15024G>A	p.C93Y	P/Possible DEAF modifier.	∼0.06	1.1	8	Probable	Tang et al. (2010)
m.15257G>A	p.D171N	P/LHON (Haplogroup J2 marker)	∼1.42	1.1	7	Unlikely	http://mitomap.org 2012; Rose et al. (2001)
m.15287T>C	p.F181L	P+Possible DEAF modifier	∼0.16	1.1	8	Unlikely	Ballana et al. (2008)
m.15315C>T	p.A190V	Polymorphism	∼0.07	1.1	5	Unlikely	Fraumene et al. (2006)
m.15452C>A	p.L236I	Polymorphism	∼10.23	15.5	7	Unlikely	Marin-Garcia and Goldenthal (1997)
m.15482T>C	p.S246P	Not present	∼0.012	1.1	5	Probable	Behar et al. (2008)
m.15813T>C	p.V356A	Polymorphism	∼0.012	1.1	7	Unlikely	Behar et al. (2008)

P, polymorphism.

1 MitoMap status, current status of the variant in the MitoMap database (http://www.mitomap.org).

### m.15024G>A, p.C93Y

**Figure 2:**
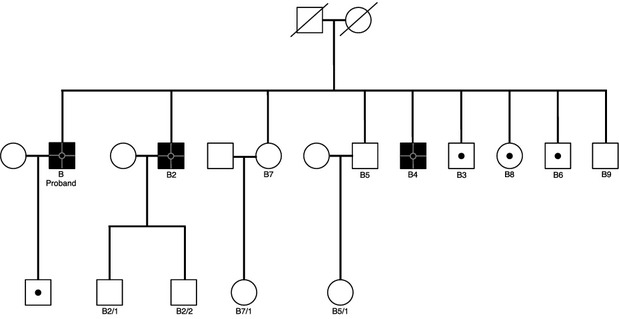
Pedigree of family B with the p.C93Y mutation. Proband B is marked with an arrow. Individuals colored black are carriers of the MHC-β p.R1712W mutation diagnosed with HCM. Individuals with a black dot are asymptomatic MHC-β, R1712W carriers.

This variant was found in a family in which a mutation, p.R1712W, in the rod domain of the beta myosin heavy chain gene (*MYH7*) had already been identified (see the pedigree in Fig. 2 and clinical information in Table 4). The variant was not found in 480 control samples. The number of family members was too small and the age differences too large to reliably perform cosegregation analysis (Table 4). The p.C93Y mutation has a low prevalence and is a marker of the very recently defined subclade F1e (Table 3). The variant has recently been suggested to be a potential as a modifier of the penetrance of a deafness mutation (Tang et al. 2010). All carriers of this variant reported here exhibited homoplasmy in blood assessed by pyrosequencing. Three of six p.R1712W carriers, all carrying the homoplasmic variant p.C93Y variant (data not shown), were symptomatic for HCM at examination at the age of 39–47 years. The sole carrier of *MYH7* p.R1712W but not *MT-CYB* p.C93Y was 14 years at the time of examination and asymptomatic for HCM. The proband was found to have a low work capacity (95 W). This is compatible with CIII-reduced activity, as exercise intolerance is a known feature of mutations affecting CIII activity (http://mitomap.org 2012).

**Table 4:** Demographic and clinical characteristics of individuals in families B and I

ID	Age (years)	BMI	Gender	Systolic BP (mmHg)	Diastolic BP (mmHg)	LVEDD (mm)	LVESD (mmHg)	LA (mmHg)	LVIVS (middle) (mm)	LVIVS (lvot) (mm)	MaxIVS (mm)	LVPWT (mm)	MaxLVD (mm)	EF (%)	LVIRT (sec)
B6	35	31	M	120	80	43	31	32	10	11	12	11	11	63	0.06
B/1	14	22	M	115	70	46	n/a	29	8	10	10	8	10	77	0.08
B	39	28	M	130	80	49	29	72	n/a	26	26	13	26	69	0.09
B2	47	29	M	140	90	47	27	40	11	13	13	10	13	80	0.07
B2/1	26	33	M	145	90	57	36	43	11	13	13	9	13	74	0.06
B2/2	24	28	M	120	80	53	37	31	10	10	10	10	10	66	0.05
B3	33	27	M	120	80	55	30	35	10	12	12	10	12	83	0.07
B4	45	31	M	130	75	47	27	40	10	13	21	11	13	83	0.08
B5/1	13	20	F	100	70	55	39	31	6	6	9	7	7	64	0.05
B7	49	36	F	130	100	45	29	42	10	11	11	10	11	74	0.06
B7/1	22	25	M	180	80	50	35	34	9	9	9	8	9	66	0.07
B9	42	26	M	130	90	53	37	36	12	12	12	12	12	64	0.08
B5	32	31	M	110	80	53	n/a	37	10	11	11	8	11	66	0.06
B8	42	28	F	150	100	45	22	36	9	8	9	9	9	88	0.07
I/2/1	16	24	M	120	60	55	43	40	8	8	8	8	8	54	0.05
I/4	39	24	M	150	80	49	31	32	10	12	12	10	12	76	0.06
I/2/2	10	20	F	100	60	38	24	22	5	5	5	6	6	77	0.05
I/2	36	24	F	100	70	46	29	34	7	7	7	8	8	75	0.08
I	64	22	F	95	60	42	n/a	35	19	27	27	10	27	n/a	0.1
I/3	36	21	F	120	80	50	35	31	6	6	6	8	8	65	0.06
I/1/2	15	19	M	95	60	47	31	34	7	7	7	9	9	72	0.06
I/1	43	24	M	120	80	50	27	35	12	11	12	n/a	12	85	0.08
I/1/1	18	21	M	120	80	50	34	31	6	6	6	8	8	71	0.05

BP, blood pressure; LA, left atrial diameter; MaxLVD, maximal left ventricular wall thickness; MaxIVS, maximal interventricular wall thickness; LVEDD, left ventricular end diastolic diameter; LVSD, left ventricular systolic diameter; LA, left atrial diameter; LVIVS, left ventricular interventricular septal thickness; LVPWT, left ventricular posterior wall thickness; EF, ejection fraction; LVIRT, left ventricular isovolumic relaxation time; n/a, the measurement was not taken in this patient.

PolyPhen predicted the p.C93Y variant to be “possibly damaging” due to overpacking at a buried site and because of its close proximity to the highly conserved, functional heme-binding site, p.H97. p.C93 is highly conserved down to the level of *Neurospora crassa* (Fig. 1). The introduction of tyrosine at position 93 in the I-TASSER-generated model of human Cytb showed that all five suggested rotamers result in substantial increases in free energy. The increased size of the tyrosine side chain was physically located toward the three transmembrane helices A, E, and B, and we hypothesized a displacement of the three helices (Fig. 7). The redox centers heme b_L_ and heme b_H_ are bound between helices A, B, C, and D; consequently, the helix displacement may result in disrupted heme binding (Hunte et al. 2000).

**Figure 3:**
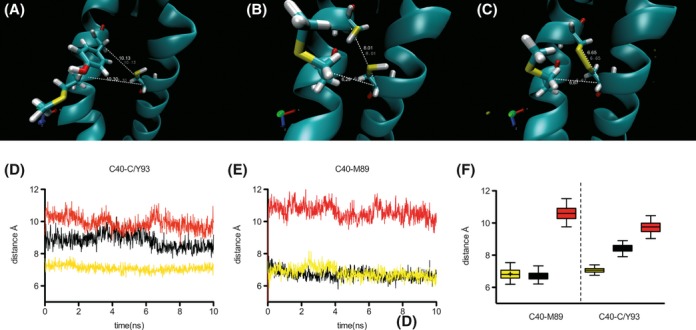
Molecular simulation of Cytb. The p.Y93 mutant is shown in red, p.C93 is shown in black, and p.C93 with a disulfide bridge to p.C40 is shown in yellow. Visualization of coordinates from the end of the simulations were generated using VMD and are shown for p.Y93 (A), p. C93 (B), and p.C93 with a disulfide bridge to p.C40 (C). The distances between p.C40 and p.C/Y93 (D) and between p.C40 and p.M89 (E) are shown as a function of simulation time. These values are shown combined (F). Whiskers are 2.5–97.5 percentile. Initial transients are discarded from p.C40-p.C/Y93 values, and data shown is from 7 ns and forward.

In order to investigate the structural effect of the mutation, we used the NAMD program and the CHARMM22 force field to simulate a 10 nsec equilibration of the system with and without the mutation, when dissolved in a water-sphere under spherical boundary conditions. In the wild-type simulation, we observed p.C40 and p.C93 aligning closely after ∼10 necs, suggesting that the proximity of the two residues is energetically favorable and indicating the possible formation of a disulfide bridge between the two residues (Fig. 3B). When we simulated the p.C93Y mutant, the mean distance between helix A and helix B increased by 3.88 ± 0.017 Å compared with the wild type, when measured between the α-carbons of the adjacent p.M89 and p.C40 (Fig. 3E and F). The structure and length of the two helices was unaltered in the mutant in the course of the simulation (data not shown). In order to test the proximity of the helices if a disulfide bridge existed, we simulated a system where the bridge is present, and found that there was little difference in mean helix displacement compared with our initial simulation of the wild-type protein (0.11 ± 0.014 Å between the α-carbons of p.M89 and p.C40) (Fig. 3E and F). If a disulfide bridge exists, the p.C93Y mutation would break this bridge and sever the covalent bond between helix A and B.

Cytb interacts with several of the 11 subunits in CIII. Neighboring residues in the connecting loops of the A, B, and E helices are important for these interactions (Berry et al. 2000). Thus, a displacement of the helices may also cause disrupted subunit interaction. The molecular modeling and simulation, the prediction by PolyPhen as possibly damaging, the recent suggestion that the variant may have a modifying role in deafness expression (Tang et al. 2010), and the clinical presentation with exercise intolerance in carriers of the variant are compatible with altered function of mutated Cytb, and supports a possible disease modifying role of p.C93Y.

### m.15482T>C, p.S246P

**Figure 4:**
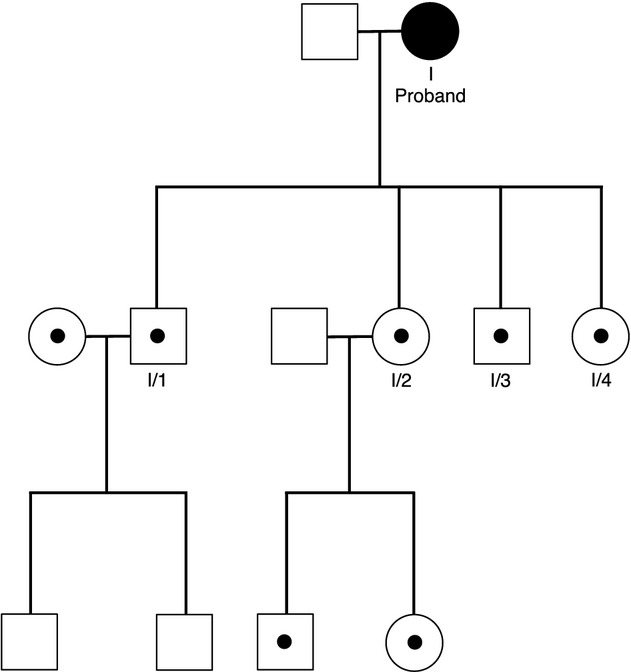
Pedigree of family I with the p.S246P mutation. Individuals with black dots are carriers of the mutation. Individuals colored black are diagnosed with HCM.

This mutation has been described in a phylogenetic context (Behar et al. 2008), has a frequency <0.1% (Table 3), and was not found in 480 control samples. The mutation was identified in a 64-year-old woman, in whom no sarcomeric mutations had been found. All carriers exhibited homoplasmy for the mutation (data not shown) (see Fig. 4 for pedigree and Table 4 for clinical information).

The serine is not highly conserved; by contrast the adjacent p.P247 is conserved in all examined Cytb sequences (Fig. 1). PolyPhen predicted the variant to be “possibly damaging” (Table 3). The introduction of a diproline severely restricts backbone conformation flexibility, which limits the options for local secondary structure (Saha and Shamala 2012). In addition, the introduction of a proline has a well-known helix-breaking effect and a proline substitution can have consequences far away from the region of the molecule in which it occurs (Yohannan et al. 2004). This type of effect has been previously documented in Cytb, where the substitution of p.K319 in yeast, equivalent to the similarly basic p.R318 in vertebrates, with proline, disrupted the geometry between two helices, thus hindering the assembly of Cytb and resulting in long-distance perturbation of the ISP headgroup (Blakely et al. 2005).

**Figure 5:**
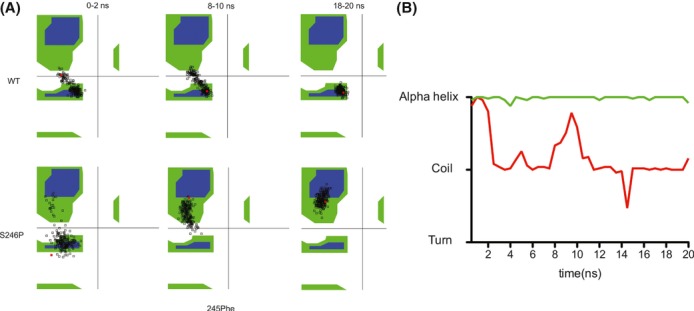
p.F245 simulated participation in secondary structure. (A) Ramachandran plots of p.F245 over the time-course of the simulation. (B) Secondary structure calculated using STRIDE during the course of the simulation is shown. Each data point on the graph corresponds to a measure of the prevalence of different secondary structures during 0.5 ns of simulation time.

**Figure 6:**
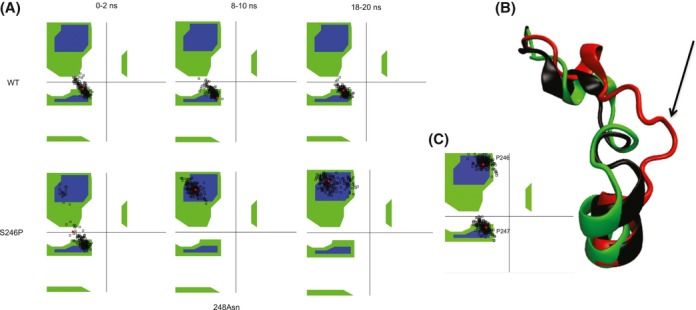
Simulated structural effects of p.S246P. (A) Ramachandran plots of p.D248 over the time-course of the simulation. (B) Final structure of p.P246 (red) compared with p.S246 (green) after 20 ns. The initial backbone of both p.P246 and p.S246 is also shown (black). The black arrow denotes the kink induced by p.P246 (C). Ramachandran plot of p.P246 and p.P247 from 18–20 ns, showing the final angle configuration, compatible with a P_II_-α_R_ conformation.

Molecular dynamics of the p.S246P mutant showed that the main effect of the mutation was a shortening of the preceding α-helix and introduction of a kink into the amino acid chain (Fig. 6B). The residue p.F245 immediately prior to the diproline changed structure with regard to the preceding residues during the course of the simulation (Fig. 5A), moving out of the area permissible for an α-helix conformation. When the secondary structure was calculated at each timepoint in the simulation using STRIDE, it was evident that p.F245 changed from a stable α-helix conformation to an unstable state that alternates between random coil and α-helix (Fig. 5B). The diproline settles into a P_II_-α_R_ conformation (φ = ∼70° for both residues, p.P246 ψ = ∼145°, p.P247 ψ = ∼−40°) (Fig. 6C), distorting the amino acid chain by introducing a kink (see Fig. 6B). p.D248, immediately following the diproline also had markedly altered backbone angles (WT: φ = ∼−55°: ψ = ∼40°, p.S246P φ = ∼−120°: ψ = ∼120°) as a result of the fixing of the backbone angles that is induced by the diproline (Fig. 6A). The general direction of the amino acid chain following the kink is conserved by steric alterations of p.G251 where the φ-angle is reversed (WT φ = −75°, P246 φ = 75°) (data not shown).

**Figure 7:**
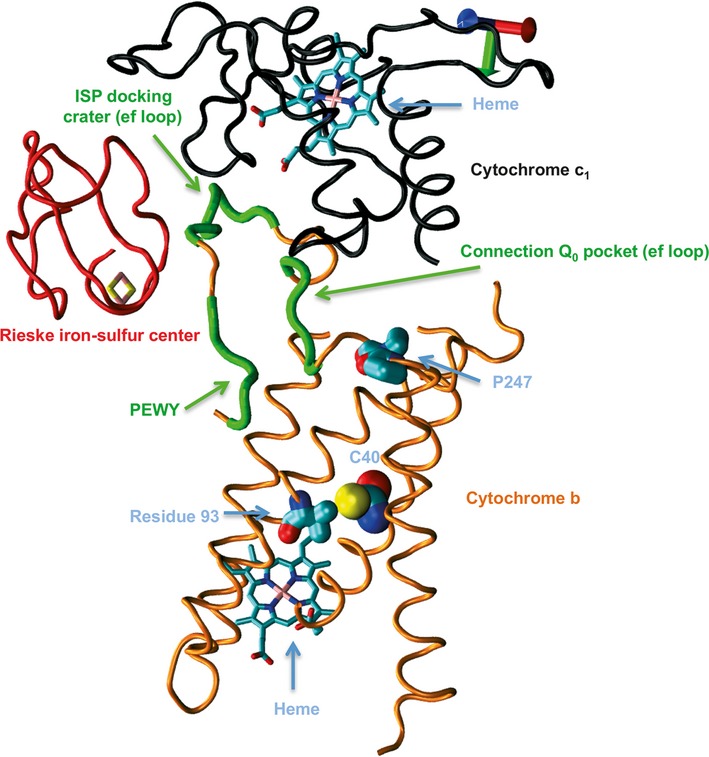
Relevant parts of yeast Cytochrome bc_1_ (PDB ID: 3cxh). Cytochrome b is shown in orange. Cytochrome c_1_ is shown in black. The Rieske subunit is shown in red. The location of the PEWY motif, ISP docking crater, and Q_0_ connection on Cytochrome b is shown in green. The location of the conserved p.P247 and the location of p.C40 and residue number 93 (valine in yeast) are shown by blue arrows. Heme groups are also marked by blue arrows.

A detailed examination using the crystal structure of CIII from yeast (pdb: 3CXH) shows that residue 246 is located at the start of the *ef* loop on the side of the membrane facing the intermembrane space (Fig. 5). The *ef* loop contains residues only 5 Å from parts of cyt C_1_ and the Rieske subunit. A change in the loop conformation could disrupt the interactions of Cytb with these subunits. The *ef* loop takes part in the conformational changes in the Q_o_-pocket domains that control the binding of the ISP (Rajagukguk et al. 2007). Residues 262 and 268 at the end of the *ef* loop forms the docking crater for the ISP, and residues 252–256 in the middle of the *ef* loop connects the Q_o_-pocket with the other two surface domains. Molecular dynamics simulations by others have shown that residues 263–268 of the *ef* loop are displaced by up to 2Å as the ISP rotates from the b state to the c_1_ state (Izrailev et al. 1999). In addition, the highly conserved PEWY motif (residues 271–274 in yeast), situated in the Q_0*_-site could be disrupted by the changed conformation as previously suggested with the p.G251D mutation (Andreu et al. 2000) (Fig. 7). On the basis of the low prevalence of the mutation and the molecular modeling and dynamics, we suggest that p.S246P has a structural significance on Cytb conformation and CIII subunit interactions, suggesting that it may be disease causing or disease modifying.

## Discussion

This study is the first systematic examination of the *MT-CYB* gene in a well-characterized cohort of HCM probands. We identified a total of 43 variants in *MT-CYB*, of which 21 were nonsynonymous. After a thorough bioinformatics analysis, we identified two rare, potentially disease-causing mutations in *MT-CYB* in 91 HCM patients. One, p.C93Y, was inherited digenically with a sarcomeric mutation, a phenomenon only rarely observed (Arbustini et al. 1998).

Interestingly, many of the rare variants we identified were unlikely to be associated with any structural effect including two variants, m.15452C>A, p.L236I and m.14927A>G, p.T61A, that have been previously reported as being associated with CIII dysfunction (Marin-Garcia and Goldenthal 1997) and cardiomyopathy (Obayashi et al. 1992; Marin-Garcia et al. 2000; Casademont and Miro 2002). m.15452C>A is a frequently occurring variant (∼4.3%) and both amino acids are weakly conserved (Fig. 1).

Mitochondrial DNA mutations can be disease causing through several mechanisms, with the most prominent being interference with OXPHOS function (Tuppen et al. 2010). mtDNA mutations can result in conditions ranging from early onset, overt life-threatening disease to slight modifications of clinical presentations of conditions of nonmitochondrial etiology (Giacchetti et al. 2004). The latter type is believed to be caused by leaky mutations, resulting in specific mild OXPHOS dysfunction leading to progressive dysfunction. Leaky mutations are not selected against, as they do not affect reproduction; they can, however, play a modifying role in multigenic diseases (Ji et al. 2012).

This is supported by the demonstration of mtDNA variants as disease modifiers and susceptibility factors (Hudson et al. 2007; Human et al. 2010). The nuclear-encoded structural genes, coding for 105 of the 118 structural subunits of the oxidative phosphorylation protein complexes, need to coadapt to the mitochondrial genes, and it is therefore likely that certain mtDNA variants are acceptable against a certain nuclear background or in the presence of mtDNA with compensating mutations (Scheffler 2008; Montoya et al. 2009). Thus, leaky mutations may become a subclade-defining variant at the tip of a branch in the mtDNA phylogenetic tree. We believe that is the case with the m.15024G>A variant which has recently been added to PhyloTree-mtDNA Tree Build 15 as a marker for the F1e haplogroup. Such a contribution to the phenotypic variability of HCM caused by mtDNA mutations may be of a more general significance.

The two putative leaky mutations identified in this study favor an altered local conformation of Cytb structure, while the general integrity of the protein remains intact. Consequently, the amino acid substitution is not severe and probably only partially disrupts the function of the protein. This in turn could result in phenotypic manifestations over time as a consequence of a lowered threshold (Rossignol et al. 2003).

The structural changes induced by the p.C93Y and p.S246P mutations may influence the stability and/or activity of the supercomplexes, comprised of CI_1_III_2_IV_1,_ modifying variation in electron leakage and/or ROS production (Sipos et al. 2003; Seelert et al. 2009). Increased ROS production has previously been shown to induce cardiomyocyte hypertrophy, apoptosis, and interstitial apoptosis (Tsutsui et al. 2009). In addition, ROS causes mtDNA damage resulting in accumulation of mtDNA somatic mutations creating a viscous circle, generating more damage, and further increasing ROS production and damage that over time could affect ATP production. Studies on transgenic mouse and rat models have shown that there is an increased tension-dependent ATP consumption associated with HCM (Xu et al. 2010). Thus, the combination of increased baseline ROS production and the concomitant lower threshold is likely to aggravate, and in some cases precipitate clinical HCM.

As our clinical material is limited and most mutation carriers are still relatively young, we can only speculate on the long-term clinical consequences of the mutations, especially because CIII isolated dysfunction may be ameliorated by compensatory mechanisms in vivo (Rossignol et al. 2003). Follow-up studies including ergometry on the patients in the coming decades will be interesting, especially in order to differentiate the clinical effect of *MYH7* p.1712W and *MT-CYB* p.93Y.

In conclusion, we find that rare, putative leaky mtDNA variants in *MT-CYB* can be identified in a cohort of HCM patients. While our data do not support an unequivocal pathogenic effect of these mutations, our in silico analysis demonstrate that the mutations do have a structural effect on protein conformation. We propose that further patients with HCM should be examined for mutations in *MT-CYB* in order to clarify the role of these rare variants. Screening of other mtDNA genes may also be beneficial as variants in several of these have already been associated with HCM.

### Limitations of the study

A limitation of this study is the scarcity of family data; in general, nuclear families are small, precluding cosegregation analysis. Furthermore, we used DNA isolated from whole blood, which, due to mitotic segregation and relaxed replication, may not be identical to the mtDNA in the cardiac tissue. On the other hand, the analysis of a large cohort makes it possible to obtain an overview of the quantitative significance of *MT-CYB* variants in HCM.
