# MT-CYB mutations in hypertrophic cardiomyopathy

**DOI:** 10.1002/mgg3.29

**Published:** 2013-09-10

**Authors:** Christian M Hagen, Frederik H Aidt, Ole Havndrup, Paula L Hedley, Cathrine Jespersgaard, Morten Jensen, Jørgen K Kanters, Johanna C Moolman-Smook, Daniel V Møller, Henning Bundgaard, Michael Christiansen

The original article to which this Corrigendum refers was published in Molecular Genetics and Genomic Medicine 1(1):54–65 (DOI: 10.1002/mgg3.5).

In Figure 4 on page 61 of the article, the pedigree shows that the family members of family I and a non-related individual (left-most circle) are carriers of the mutation. The authors realized that there was an error in the figure, in that the non-related individual is not a carrier. Therefore, this error has been corrected in the new Figure 4 (see below), by removing the dot in the left-most circle.

**Figure 4:**
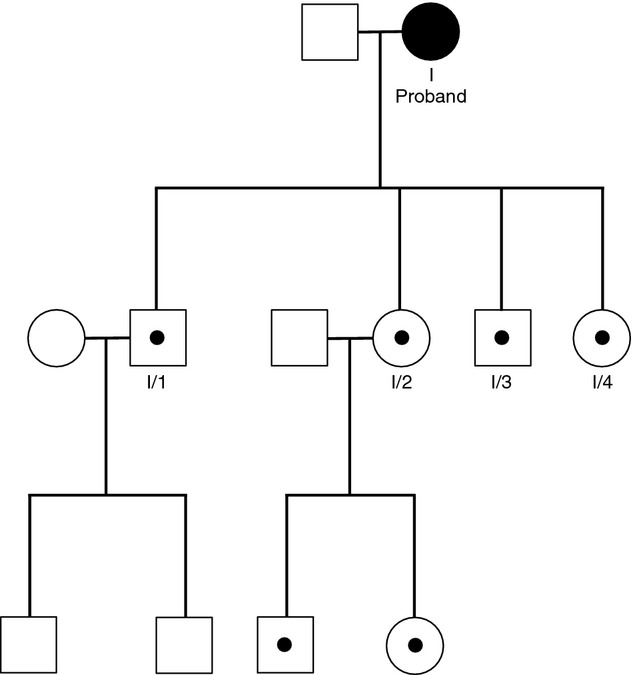
Pedigree of family I with the p.S246P mutation. Individuals with black dots are carriers of the mutation. Individuals colored black arediagnosed with HCM.

The authors apologize for this oversight.

